# Tissue distribution, placental transfer and excretion of silver nanoparticles in pregnant rats after a single oral dose

**DOI:** 10.5620/eaht.2023023

**Published:** 2023-11-14

**Authors:** Khaled Y. Abdel-Halim, Elsayed I. Salim, Ahmed S. Abdel-Latif, Sally E. Abu-Risha

**Affiliations:** 1 Mammalian & Aquatic Toxicology Department, Central Agricultural Pesticides Laboratory, Agricultural Research Center, Giza, Egypt; 2 Tanta University, Faculty of Science, Department of Zoology, Research Lab. of Molecular Carcinogenesis, Tanta, Egypt; 3 Tanta University, Faculty of Pharmacy, Department of Pharmacology & Toxicology, Tanta, Egypt

**Keywords:** Silver nanoparticles, Pregnant rats, Excretion, ICP-OES, Tissue distribution, Placental transfer

## Abstract

A quantitative assessment of silver nanoparticles (AgNPs) in fluids and some organs of pregnant rats as well as their fetal blood were carried out in this study. A single oral dose (1mg/kg) of AgNPs with a size range of 4-20 nm was administered to pregnant rats on the 19th of gestation. Five groups were euthanized after 10 min, 1, 6, 12, and 24 hr as well as the control group. Total Silver (Ag) contents were measured in bloods (maternal and fetal) and several organs using Inductive Coupled Plasma Optical Emission Spectroscopy (ICP-OES) followed by acid digestion. In maternal blood, AgNPs were found to increase time-dependently after 12 and 24 hr into 0.135 and 0.224 μg/ml, but it was slightly higher in fetal blood (0.32 and 0.31 μg/ml) after 10 min and 1 hr. In other samples: kidneys, liver, spleen, placenta, and uterus the data indicated that NPs were rapidly absorbed from the dosing site (gastrointestinal tract) as evidenced by the detection of Ag in the analyzed samples (fluids and tissues). On the other hand, the cumulative percentages of excretion level in urine was 8.25% which was higher than in feces (4.77%) after 24 hr. These findings indicate the ability of AgNPs to accumulate in pregnant rats and transfer to their fetus imposing adverse outcomes and male formation. Thus, further investigations must be followed for direct and/or indirect exposure to such NPs before decision for their practices.

## Introduction

Silver nanoparticles (AgNPs) were used as antimicrobial coatings in medical devices. Also, it can be used in many medical applications such as wound dressings, silver-impregnated fabrics for clinical clothing, antimicrobial packing paper for food preservation, cardiovascular implants, dental materials as well as bio-diagnosis. Moreover, they have a broad spectrum of applications e.g., air disinfection, drinking water purification systems, and food packaging [1].

Nanotechnology is a hurriedly developing and growing field leading to an increase of engineered nanomaterials with practically new physical and chemical properties, which might induce hazardous effects in biological systems [2]. Therefore, inclusive knowledge about possible toxicologic pathological effects of nanoparticles (NPs) in biological systems is crucial regardless of whether NPs exposure is intended or not. The growing number of profitable supplies and the widespread of NPs application areas increase concerns about NPs accumulation and long-term retention in organisms and subsequent toxicological effects. The emerging science of nano-toxicology is a newly developing discipline as a part of bionanotechnology sciences. It was recently imposed in industrial countries after the high increase tendency of using nanotechnology. Mammals, especially humans are exposed to NPs which can penetrate into the body with inhalation, ingestion, and dermal. So, a lot of work concerning toxicological effects was done. These NPs are able to penetrate the body in a transcutaneous manner. The size of NPs permits endocytosis to penetrate a cell and transcytosis to penetrate several cells, resulting in uptake in shallow and/or deep compartments e.g. bone marrow, lymph nodes, fetuses, uterus, and others. Such absorption can induce adverse effects. For example, the prospective fetal toxicity depends on the passage of chemical particles from the maternal fluids which are mainly determined through the placental barrier in the case of mammals [3].

The placenta has traditionally been regarded as a barrier preventing the passage of harmful substances, which protects the embryo and fetus from toxic exposure. Indeed, the placenta has a number of proteins, which are involved in the transport of unworked substances back to maternal circulation as well as in retaining and detoxication of toxic substances. However, accidental exposures in pregnant women have made it evident that the placenta cannot prevent the passage of teratogens or metals [4]. In fact, adverse pregnancy outcomes (low birth weight, prematurity and intrauterine growth retardation) are associated with exposure to xenobiotics. These effects could be related to changes in the functional morphology of the placenta. One of these xenobiotics is metallic NPs, where several studies demonstrated placental transfer of NPs in rodents e.g. gold NPs (AuNPs) [5], oxide-bused particles, fullerenes (C60), carbon-black and titanium dioxide NPs (TiO2NPs) [6], and fluorescent quantum dots (QDs) [7]. This study was designated to explore the accumulation levels and excretion of AgNPs in organs and body fluids in both mothers and fetuses in relation to their placental transfer.

## Materials and Methods

### Chemicals

Highly purified AgNPs colloids suspended in 0.9% trisodium citrate solution were obtained from Nano Laboratories, Dream City-6-October, Cairo, Egypt. Nitric acid (HNO3; 69%) and hydrochloric acid (HCl; 30/34%) were supplied by SDFEL Co., Industrial Estate, Mumbai, India. Hydrogen peroxide (H2O2; 30%) was supplied by Central Drug House (P) Ltd. New Delhi, India. Type 1 Milli-Q deionized water obtained from the Central Laboratory of Tanta University, Egypt (Ultrapure water purification system, Merck-Millipore ) was used in all experimental procedures.

### Characterization of AgNPs

#### UV-VIS Spectrophotometric analysis

Pure Ag+ ions in an aqueous solution were detected by the sampling of aliquots (0.2 ml) of colloidal suspension, then diluted into 2 ml of ultrapure Milliq deionized water and subsequently measured by using UV-VIS spectrophotometer (LABOMED model UVS, USA) at the Central Laboratory of Tanta University. The absorption pattern was scanned at wavelengths ranging from 350 to 600 nm.

#### Transmission Electron Microscopy (TEM)

This analysis was employed to visualize the size and shape of AgNPs. The colloidal material was suspended in an ultrasonic water bath for 50 min and an aliquot of AgNPs was placed on the carbon-coated copper grids after allowing the water to evaporate drying in the air. The electron micrographs were then taken by analyzing the prepared grids on JOEL-TEM Instrument (JOEL 100 SY, Japan).

### Placental transfer experiment

#### Animal husbandry and mating

The study was performed on pregnant Wister rats. The institutional animal care and use committee of the Faculty of Science, Tanta University, Egypt, approved the animal caring ethics (No. IACUC:SCI:TU:003). The guidelines for animal care were followed (NIH Publications No. 8023, revised 1978). The animals were allocated to plastic cages covered with metal grids with dry husk pudding and allowed to acclimate for 1 week in the animal facility conditions, before dividing into groups for experimentation. Animals had free access to food and water at the animal facility. Female rats were housed with males in groups of two females with one male in each cage and allowed for mating.

#### Animal treatment

On the 19th day of gestation, pregnant rats weighing an average of 192 ± 10 g were given a single oral dose of 1 mg/kg which is equal to 2 times the No Observed Adverse Effective Level (NOAEL) of AgNPs [8]. The untreated group was administered an equal volume of the vehicle. Twenty-five female rats were treated by AgNPs, and then dissected at the following time intervals; 10 min, 1, 6, 12, and 24 hr. Five animals were used at each time point [9].

All rats were sacrificed at each time point and were subjected to an excess of desflurane anesthesia. Three normal control non-treated pregnant rats were dissected at each corresponding time point. Blood samples were withdrawn from the dorsal aorta using syringes containing EDTA solution and then prepared for analysis. Maternal organs: kidney, liver, spleen, placenta, uterus as well as fetuses were collected, and stored at -20 °C until analyzed. Also, amniotic fluid, feces, and urine samples were collected as described above.

#### Quantification of AgNPs accumulation

Silver (Ag+) ions were extracted from the kidneys, liver, spleen, placenta, uterus, maternal blood, fetal blood, amniotic fluid, feces, and urine by acid digestion process according to the method of Jimenoz-Lamana et al. [10] with some modifications. One g of each sample was digested with 5 ml of HNO3 during ultrasonic radiation until a clear solution, with adding 0.5 ml of HCl. Two hundred µ l of H2O2 were added to improve the digestion process. The mixture was diluted, filtered, and completed to 30 ml with deionized water.

Total Silver (Ag) content was measured on Inductively Coupled Plasma Optical Emission Spectrometer (ICP-OES; Optima 700 Perkin Elmer, USA). An autosampler was used to deliver samples in an instrumental cyclonic spray chamber with a mass flow-controlled laser nebulizer with a gas flow of 0.65 L/min. The instrument was operated in a fast-sequential mode and featured cooled CCD detection.

The limit of detection (LOD) of Ag was calculated as double the standard deviation of a series of measurements of a solution against the blank absorbance to be 0.5 ppb. Samples were carefully handled to avoid contamination. A recovery experiment was carried out by spiking untreated samples with 100 µ g of NPs and processing them as described above. The average recoveries were 82% for blood and body fluids and 66% for animal tissues.

### Statistical analyses

All data were presented as Mean ± SE and subjected to analysis of variance (ANOVA), where values were considered significant at P<0.05. The statistical analysis was performed using the Costat program [11].

## Results

### AgNPs characterization

**Figure 1.**
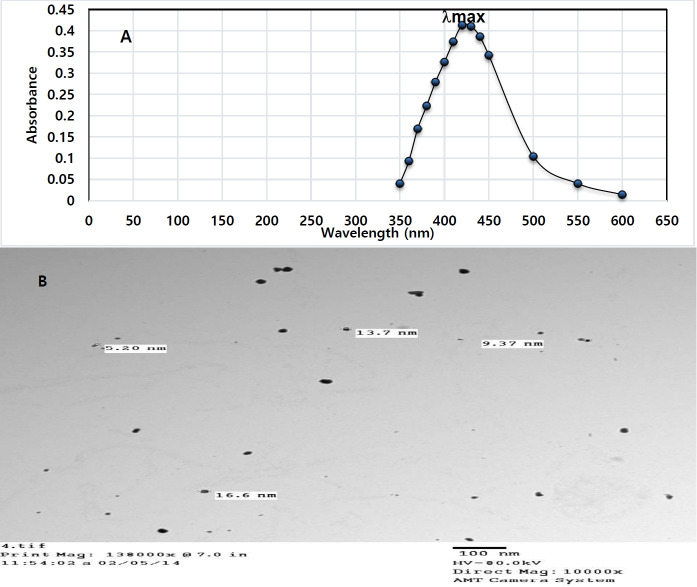
(A) UV-VIS spectrophotometric scan of AgNPs in the range of 200-650 nm; (B) TEM image visualized at 10000X of suspended AgNPs in 0.9% trisodium citrate solution.

Nanoparticle (NP) characteristics were analyzed by UV-VIS spectrophotometer and TEM (Figure 1 a-b). The maximum absorbance ( max) was detected at 420 nm, which ensures the presence of AgNPs in the suspension as previously stated by Lubick [12]. However, TEM images showed a majority of nearly spherical NPs in the range of 4-20 nm.

### AgNPs accumulation data in different tissues

#### Blood

In maternal blood, the Ag concentrations were below detection limits (BDL) after 10 min of oral gavage. However, Ag levels exhibited 0.088 and 0.082 µ g/ml after 1 and 6 hr, respectively, followed by higher levels of 0.135 and 0.224 µ g/ml after 12 and 24 hr. On the other hand, Ag levels in fetal blood were slightly higher than those detected in maternal blood, with arising values of 0.32 and 0.31 µ g/ml after 10 min and 1 hr, respectively. The highest value (3.67 µ g/ml) was recorded after 6 hr. After 12 and 24 hr, Ag contents in fetal blood gradually declined with time into 0.23 and 0.08 µ g/ml, respectively. Sum Ag content in both maternal and fetal blood combined together revealed the highest value (3.752 µ g/ml) after 6 hr, while most data were almost constant at the different sacrifice time points (Table 1).

#### Organs

**Figure 2.**
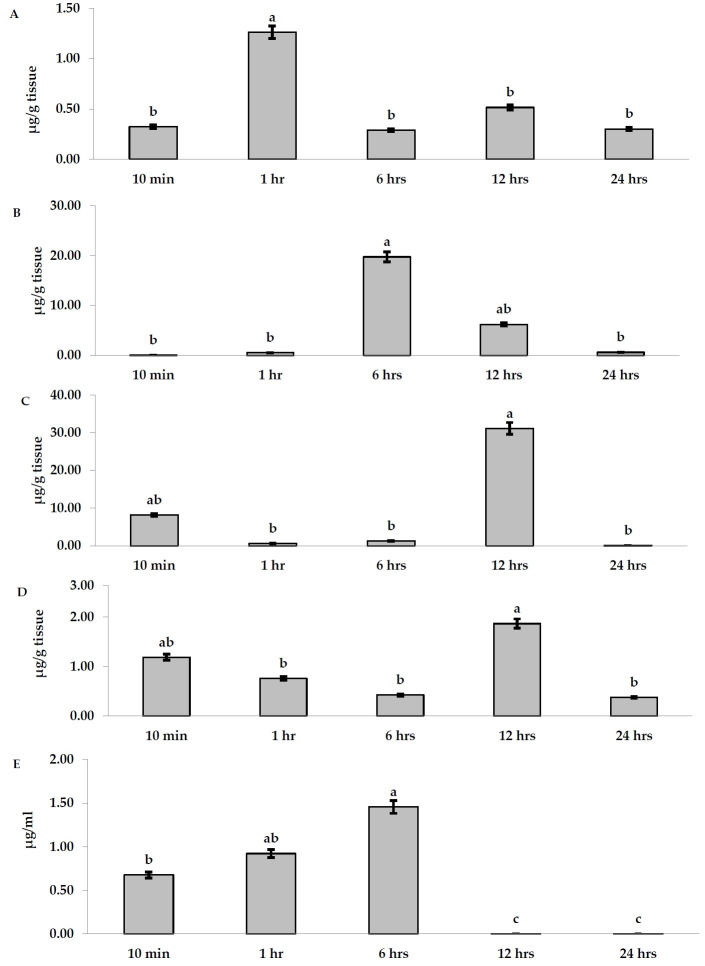
AgNPs accumulation levels (µ g/g tissue) in (A) placenta; (B) uterus; (C) kidney; (D) liver; and (E) amniotic fluid in the pregnant rats given a single i. g dose at different time points. Different letters denote significance at P<0.05.

In placental tissues, Ag levels exhibited the highest value (1.26 µ g/g tissue) after 1 hr. No statistical differences were noted during other time points, where Ag levels were as follows: 0.32, 0.29, 0.51, and 0.30 µ g/g tissue after 10 min, 6, 12, and 24 hr, respectively (Figure 2a). In uterus tissues, no Ag ions were detected after 10 min, while they exhibited 0.54 µ g/g tissue after 1 hr. After 6 hr, a sharp increase in Ag levels was indicated (19.76 µ g/g tissue). Afterward, this increase was time-dependently declined after 12 hr to 6.21 µ g/g tissue, followed by 0.61 µ g/g tissue after 24 hr (Figure 2b). Kidney tissues exhibited high levels of Ag (8.196 µ g/g tissue) after 10 min. The accumulation of Ag significantly declined (0.60 and 1.28 µ g/g tissue) after 1 and 6 hr, respectively. However, it reached the greatest value (31.136 µ g/g tissue) after 12 hr, followed by BDL after 24 hr (Figure 2c). The concentration of Ag in liver tissues was found constant and almost similar between groups after 10 min, 1, 6, and 24 hr without intergroup significant differences, except increased level which was detected after 12 hr (Figure 2d).

The concentrations of Ag in amniotic fluid were gradually increased by time being 0.675 µ g/ml after 10 min, 0.921 µ g/ml after 1 hr, and 1.457 µ g/ml after 6 hr, respectively. Interestingly, Ag levels were found BDL after 12 and 24 hr (Figure 2e).

#### Placental transfer of AgNPs

**Figure 3.**
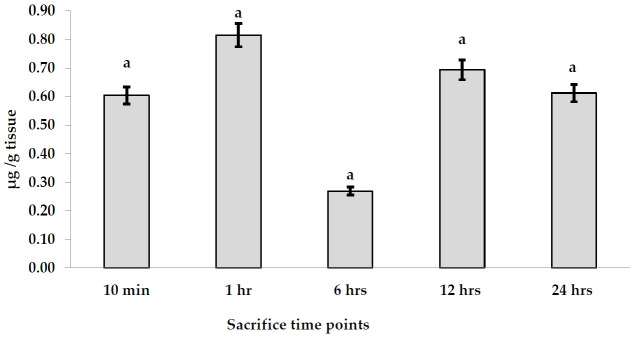
AgNPs accumulation levels (µ g/g tissue) in fetus tissues after a single oral dose at different time points.

The accumulation of AgNPs was found in the fetuses per g tissues after a signal oral gavage to pregnant rats as illustrated in Figure 3. The accumulation of AgNPs in fetal tissues exhibited the highest value (0.815 µ g/g tissue) after 1 hr, while the least one (0.269 µ g/g tissue) was detected after 6 hr. In contrast, AgNPs were redistributed after 12 and 24 hr, accounting for 0.694 and 0.613 µ g/g tissue, respectively. On the other hand, the mean values of the total recovered amount of embryonic Ag concentration in fetal blood, placenta, uterus, amniotic fluid, and the whole fetus was increased from 1.923 µ g/g tissue after 10 min to 3.85 µ g/g tissue after 1 hr. It reached a peak after 6 hr (25.45 µ g/g tissue). After that, AgNP concentrations recovered in the whole embryo started to decline time-dependently to 7.651 and 1.603 µ g/g tissue after 12 and 24 hr, respectively.

**Figure 4.**
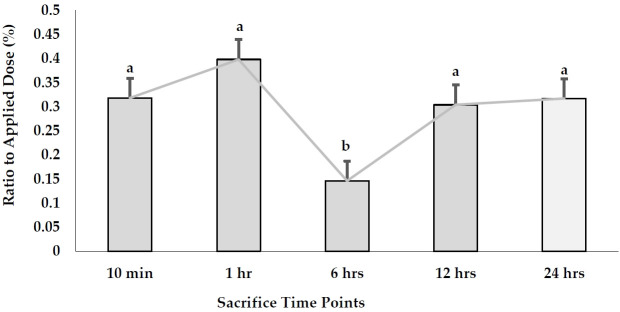
Placental transfer levels of AgNPs (%) in the pregnant rats given a single oral dose at different time points.

Moreover, percentage of AgNPs values transferred to the fetus in relation to the applied dose (% transfer) were calculated at each time point (Figure 4). The data stated that the placental transfer of AgNPs to the fetus was almost constant in all time points, except that at the 6 hr point which exhibited a significantly low value (0.146%) of the applied dose (P<0.05).

#### AgNPs excretion

**Figure 5.**
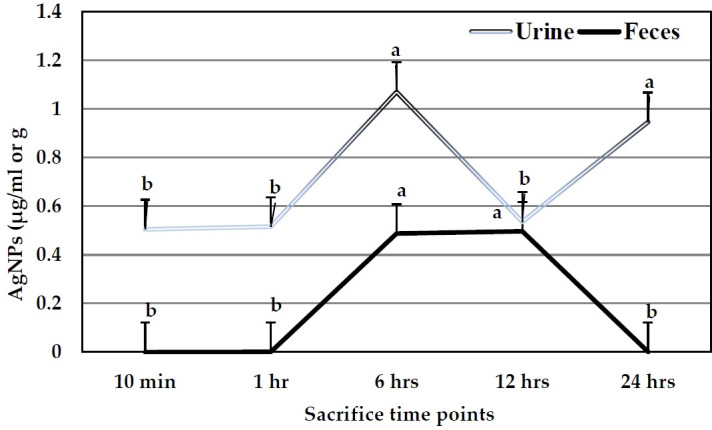
AgNPs concentrations in urine (µ g/ml) and feces samples (µ g/g) of the pregnant rats after a single oral dose at different time points.

The excretion levels of AgNPs in urine and feces are illustrated in Figure 5. The concentrations of AgNPs detected in urine samples were higher than those indicated in feces samples. The levels of Ag exhibited the highest value (1.07 µ g/ml) in urine after 6 hr, while the last one was 0.51 µ g/ml after 10 min. No significant differences were noted when levels of AgNPs were compared between 10 min, 1, and 12 hr time points. In the case of feces samples, AgNPs exhibited the highest concentration (0.496 µ g/g tissue) after 12 hr, followed by 0.487 µ g/g tissue after 6 hr, while other time points exhibited no detected values.

**Figure 6.**
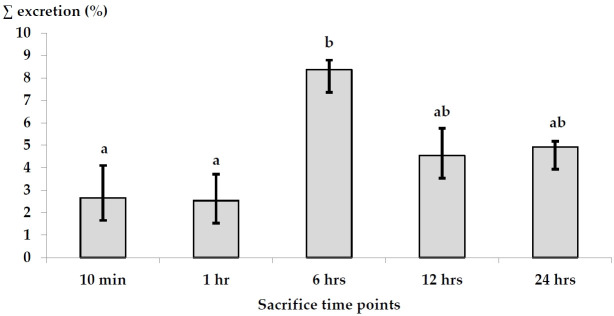
Sum of the excretion percentages of AgNPs in both urine and feces samples collected from the pregnant rats given a single oral dose.

Cumulative excretion levels of AgNPs in both urine and feces in relation to the applied dose at all the time points were higher in urine (8.25%) than those in feces (4.77%) after 24 hr. In urine, the highest excretion percentage was 5.79% after 6 hr, followed by 4.92% after 24 hr. No significant differences were observed at other time points. On the other hand, the excretion levels in feces samples exhibited the highest value (2.57%) after 6 hr, followed by 2.18% after 12 hr. Sum excretion in both urine and feces exhibited the highest value after 6 hr (Figure 6).

## Discussion

The ability of AgNPs to distribute, penetrates, accumulate, and be excreted in pregnant rats as well as their transplacental transfer to fetuses was assessed here. The examined NPs were successfully prepared, and characterized, where the NP size ranged between 4-20 nm. The data obtained from organ accumulation of AgNPs indicate that they are rapidly absorbed from the dosing site (gastrointestinal tract) as evidenced by the detection of Ag in all analyzed organs and fluids. Moreover, the accumulation of AgNPs in the placenta during all sacrifice time points was lower than in the other organs or surrounding components such as the uterus and amniotic fluid at certain time points. The likelihood of such transport of various types of NPs has repeatedly been postulated as a potential source of risks to the development of fetuses and newborns [13, 14]. The present data are in agreement with that obtained by Yang et al. [15], where AgNPs were identified in the fetuses of all pregnant rats in amounts significantly exceeding the detection limits after intragastric administration. Also, it was reported that differently sized silica dioxide SiO2 and TiO2NPs showed accumulation of fluorescently labeled 70 nm of SiO2 and 143 nm of TiO2 in the placenta after 24 hr of i. v injection [16]. However, the present work has novelty for AgNPs tissue distribution and excretion patterns in association with its placental transfer and fetal uptake.

The placenta has an active and selective role in drug and chemical transfer mechanisms. On these bases, the placental transfer would occur essentially through its semi-permeable membrane determined by physical characteristics such as the thickness of the membrane, pore size, molecular weight of a substance on another side of the membrane, perfusion pressure (maternal blood pressure), and placental flow [17]. The diffusion process depends also on molecular size and spatial configuration, ionic dissociation, and liquid solubility substance [18]. In the present study, the AgNPs accumulated in the whole body of fetuses were higher than the remaining NPs in placental sections at different time points indicating a transfer of NPs to the fetus and amniotic fluid. We have previously shown that AgNPs induced placentalhistopathological lesions, 8-OHdG genotoxicity, and fetal cytotoxicity after i. v single administration in pregnant rats [9].

There are many investigations stating the transplacental passage of NPs in rodents. For example, gold nanoparticles (AuNPs) sized 1.4 and 18 nm were recorded to induce placental transfer in female rats after i. v injection [19]. Their uptake in the placenta was 3 and 0.02% for 1.4 and 18 nm-sized NPs, respectively. Another study on the same NPs by Yang et al. [20] demonstrated that NPs at the size range of 12-14 nm were found in mouse fetuses following i. v administration to pregnant mothers. In another investigation, SiO2NPs of a bigger size, 300 or 1,000 nm were not observed in the placenta or fetuses [16]. The ability of 50-100 nm in diameter fluorescent polyester NPs to penetrate the fetoplacental barrier modeled by a monolayer of human choriocarcinoma cells was demonstrated by Cartwright et al. [21]. Penetration of CdSe quantum dots (QDs) via fetal placental barrier after parenteral administration to female mice was described [22].

Among transplacental mechanisms, previous studies indicated that NPs transport highly depends on NPs size [23]. The threshold of NPs penetration through the barrier was found to be between 143-300 nm for SiO2NPs in mice [16] and between 80-240 nm for polystyrene NPs in humans [24]. In situ-formed human plasma protein corona was investigated by Gruber et al. [25] in relation to the movement of 80 nm polystyrene nanoparticles (PS-particles) across the human placenta. They showed that the PS corona dramatically and dynamically changes once it crosses the human placenta and that the initial makeup of PS particles in the mother's blood does not predict how well they will transfer or function after transfer. However, many authors indicated that the glomerular filtration threshold for NPs is between 6-8 nm [26] which is lower than that for the placental barrier. In addition to these concepts, it was established that NPs transplacental passage depends on the size and coating of QDs, where the application of smaller QDs (1.7 nm compared to 2.6 and 3.2 nm) results in higher Cd levels in tissues. Additionally, when the particles were stabilized with a SiO2 or polyethylene glycol (PEG) coating, the transfer level was noticed to be decreased [27].

To study AgNPs excretion, its accumulation in both urine and feces was assayed in the present study. As described above, the percentages of urine excretion were higher than those of feces, recording the highest value after 6 hr of administration. This concept is in agreement with that obtained by Loeschner et al. [28], where in studies using i. v dosing it was documented that biliary excretion of Ag was high and against a plasma-to-bile concentration gradient, where the concentration in bile was 16 to 20 times higher than in plasma from rats. On the other hand, oral gavage of AgNPs stimulated more absorption of NPs on non-digestive food components and increased their excretion in feces more than in urine [28]. Furthermore, the binding of silver to the intestinal surface could result in a decreased excretion of metal in feces [29].

In fact, the transplacental NPs to fetuses may represent adverse outcomes, especially arising malformation, the decreased weight of births, and others. Most toxicological effects of NPs in organisms were stated in the literature sites. As mentioned previously, Asharani et al. [30] showed that AgNPs can enter the mitochondria and nucleus besides the cytoplasm of different cells. Their localization in these cellular components may induce disorders in cellular function [31, 32]. The localization of AgNPs in mitochondria and nucleiimposes a good marker among NP’s genotoxic effects on organisms. This induction exactly depends on the ability of AgNPs to liberate Ag which can induce cell-specific, localized cellular toxicity as suggested by the Trojan horse theory [33]. Additionally, their ability to form ROS is considered the main pathway for NPs genotoxic effects and other risks. Increased Reactive Oxygen Species (ROS) level is related to a series of events leading to membrane damage, DNA and protein damage, and apoptosis or necrosis [18].

A number of previous studies employing various kinds of cultured cells and animal models suggested that both genotoxicity and apoptosis are important mechanisms for AgNPs induced toxicity. For example, AgNPs induced DNA breakage in some cell lines using DNA comet assay [31, 34], DNA double-stranded breaks in treated human hepatoma cells [35] and mouse embryonic fibroblasts and stem cells [36]. In research on gene expression using oxidative stress and antioxidant defense polymerase chain reaction array, the expressions of 17 of the 59 genes on the arrays were altered in cells treated with AgNPs. These genes are involved in producing ROS, antioxidants, and oxygen transporters, and in oxidative stress responses and DNA repair. It was also suggested that 5 nm AgNPs are mutagenic in mouse lymphoma cells owing to the induction of oxidative stress [37].

## Conclusions

The present work conducted the distribution, excretion and fetal uptake of AgNPs after a single dose in the pregnant rats. Such this finding indicates the predicted risk association with direct and/or indirect exposure to AgNPs. In fact, the increase and widespread use of AgNPs is of increasing concern with regard to the safety of human health, especially in consumed products and medical applications. So, more toxicological pathological investigations must be done on AgNPs or engineered NPs to establish their limits of regulation and margins of safety to mammals, before practice decision.

## Figures and Tables

**Table 1.** Concentrations of AgNPs (μg/ml) in maternal and fetal blood of the pregnant rats after a single oral gavage.

Sacrifice time point(hr)	AgNPs (μg/ml)		
	Maternal	Fetal	ΣMean
10 min	BDL	0.32 ± 0.02^b^	0.32 ± 0.02^b^
1	0.088 ± 0.01^b^	0.31 ± 0.04^b^	0.39 ± 0.05^b^
6	0.082 ± 0.12^ab^	3.67 ± 0.17^a^	3.75 ± 0.16^a^
12	0.135 ± 0.05^ab^	0.23 ± 0.13^b^	0.37 ± 0.18^b^
24	0.224 ± 0.05^a^	0.080 ± 0.04^b^	0.30 ± 0.02^b^
Control	BDL	BDL	BDL

- Each value is the mean of 5 animals ± SE. - The same letters indicate no significant differences at 0.05 levels - BDL= below the detection limit
